# Signal duration and the time scale dependence of signal integration in biochemical pathways

**DOI:** 10.1186/1752-0509-2-108

**Published:** 2008-12-17

**Authors:** Jason W Locasale

**Affiliations:** 1 Department of Biological Engineering, Massachusetts Institute of Technology, 77 Massachusetts Ave., Cambridge, MA 02139, USA; 2 Department of Systems Biology, Harvard Medical School, Division of Signal Transduction, Beth Israel Deaconess Medical Center, Boston MA 02115, USA

## Abstract

**Background:**

Signal duration (e.g. the time over which an active signaling intermediate persists) is a key regulator of biological decisions in myriad contexts such as cell growth, proliferation, and developmental lineage commitments. Accompanying differences in signal duration are numerous downstream biological processes that require multiple steps of biochemical regulation.

**Results:**

Here we present an analysis that investigates how simple biochemical motifs that involve multiple stages of regulation can be constructed to differentially process signals that persist at different time scales. We compute the dynamic, frequency dependent gain within these networks and resulting power spectra to better understand how biochemical networks can integrate signals at different time scales. We identify topological features of these networks that allow for different frequency dependent signal processing properties.

**Conclusion:**

We show that multi-staged cascades are effective in integrating signals of long duration whereas multi-staged cascades that operate in the presence of negative feedback are effective in integrating signals of short duration. Our studies suggest principles for why signal duration in connection with multiple steps of downstream regulation is a ubiquitous motif in biochemical systems.

## Background

**Figure 1:**
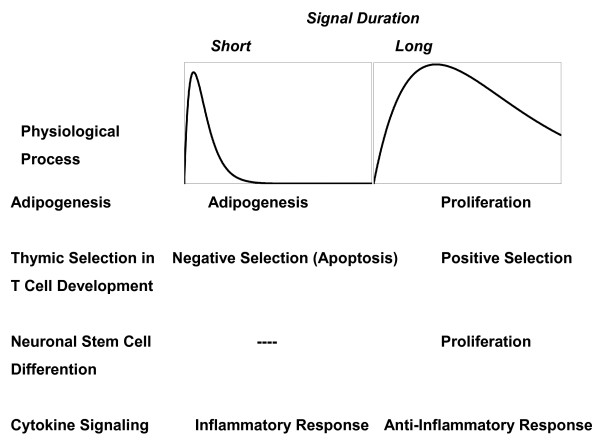
**Physiological examples of signal duration determining the phenotypic outcome in signal transduction**. Four examples of physiological processes in which branching phenotypic decisions are believed to be controlled by the detection of differences in signal duration [5,6,8,28-30].

Signal duration (e.g. the length of time over which a signaling intermediate is active) is a critical determinant in mediating cell decisions in numerous biological processes including cell growth, proliferation, and developmental lineage commitments (Fig. 1) [1-8]. One fundamental issue in signal transduction and cell decision making then is how differences in signal duration are detected to achieve the appropriate biological response.

Accompanying changes in signal duration are multiple stages of biochemical regulation of differing network topology that collectively integrate an incoming signal to deliver a specific biological response. The sequential activation of multiple steps in a biochemical pathway is a ubiquitous regulatory motif involved in many aspects of gene regulation, metabolism, and intracellular signal transduction. Many advantages of having multiple steps of regulation as opposed to having activation occur through a single step have been documented. A signaling cascade can allow for attenuation of noise, incorporation of additional regulatory checkpoints or proofreading steps, and increased tunability of the input signal [9-12]. Other studies have established conditions under which signaling cascades amplify or attenuate incoming signals [13-15]. These conditions are established by rates of activation, rates of deactivation that are set by phosphatase activities, and the presence of scaffold proteins [14-16]. However, how these features synergize with downstream effector pathways to detect differences in signal duration has not been fully studied.

A recent study has proposed a model that predicts how signals with different dynamical characteristics can be distinguished upon integration into different network architectures [17,18]. We develop a formalism to complement their approach and, as a consequence, identify general principles for how different network topologies can differentially integrate signals that persist at different time scales. We focus on a simple model of the sequential enzymatic activation of multiple species along a pathway to understand mechanistic principles underlying how multiple stages in a biochemical pathway can integrate differences in signal duration. We use a model of a weakly activated cascade[13,14,19], whose assumptions we first motivate, to study the question of how biochemical cascades detect the time scale dependence of input signals. Our approach is similar to previous work [20] that investigated the frequency dependent signal processing properties of single enzymatic cycles. The model allows us to characterize the dynamics by obtaining exact expressions for the power spectra of linearized biochemical networks of multiple stages with arbitrary length and connectivity and we focus on how these frequency dependent signal processing properties of cascades are used to detect differences in signal duration.

We first show that biochemical cascades can function as both high low and high pass filters depending on the topology of the network architecture. A low pass filter removes high frequency (short duration) components of a signal and a high pass filter removes low frequency (long duration) components of a signal. These filtering capabilities are determined by differential positive and negative regulation within the biochemical pathway. Importantly, the filtering capabilities are determined by the presence of feedback as well as the amplification and attenuation properties at different steps in the cascade that are set by the differences in phosphatase activities at different stages along the cascade. Ultimately, our findings suggest design principles that characterize how biochemical cascades are well suited for detecting time scale dependent differences in biochemical signals.

## Results

### Detection of long duration signals

**Figure 2:**
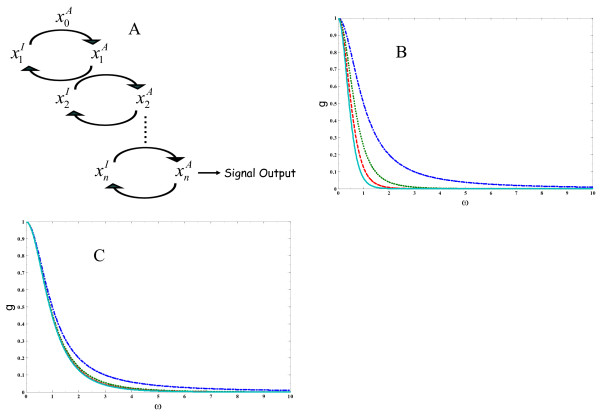
**Filtering of high frequency signals**. Time dependence of signal integration in a linear biochemical cascade. a.) the sequential activation of multiple stages in a signaling cascade. Superscripts (I) and (A) denote inactive and active forms of each chemical species and are dropped from the equations in the text. b.) same kinetic constants, all kinetic constants are taken to be: $k_{i}^{+}=k_{i}^{-}=1.0$ c.) a positive gradient of activation/deactivation rates keeping $\frac{k_{i}^{+}}{k_{i}^{-}}=1.0$ fixed. $k_{1}^{+}$ = 1.0, $k_{2}^{+}$ = 3.3, $k_{3}^{+}$ = 6.6, $k_{4}^{+}$ = 10.0. c.) plots of *g*_*n*_(*ω*) for *n *= 1, 2, 3, 4 with successively different values of $k_{i}^{-}$ while keeping $\frac{k_{i}^{+}}{k_{i}^{-}}=1.0$ fixed ($k_{1}^{-}$ = 1.0, $k_{2}^{-}$ = 3.3, $k_{3}^{-}$ = 6.6, $k_{4}^{-}$ = 10.0).

In the model as previously developed[14], an input signal, *f*(*t*), activates the first member of the pathway whose activation can then activate the next member. In turn, each upstream species activates its immediate downstream target and can also be deactivated by, for example, a phosphatase. Assuming Michaelis Menten kinetics for the activation and deactivation of each species along the cascade, we can write an equation for the dynamics of the active form of the *i*^*th *^species *x*_*i *_along the cascade:

(1) $$\frac{dx_{i}}{dt}=\frac{k_{cat}^{i}x_{i-1}\left(x_{i}^{T}-x_{i}\right)}{K_{M}^{i}+\left(x_{i}^{T}-x_{i}\right)}-\frac{k_{cat,pase}^{i}E_{pase}x_{i}}{K_{M,pase}^{i}+x_{i}},$$

where *E*_*pase *_is the concentration of the enzyme that deactivates species *i*, $k_{cat}^{i}$, $k_{cat,pase}^{i}$ are the catalytic constants of the activation and deactivation steps, $k_{M}^{i}$, $k_{M,pase}^{i}$ are the Michaelis constants and $x_{i}^{T}$ is the total amount species available at step i. We take $k_{M}^{i}$, $k_{M,pase}^{i}$ at each step to be large ($k_{M}^{i}$, $k_{M,pase}^{i}$ > $x_{i}^{T}$) so that the kinetics of the reactions are not limited by the availability of the enzyme [21]. This assumption implies that the enzyme kinetics operate in a linear, first-order regime. Next, we assume that the cascade is weakly activated (i.e. at each stage, the total number of species is much larger than the number of active species, $x_{i}^{T}$ >> *x*_*i*_). In many biologically relevant instances (e.g. the Mitogen activated protein kinase (MAPK) cascade), the neglect of saturation effects is often reasonable [22]. Further, modeling the deactivation as a first order reaction is often valid when phosphatases are in excess as is the case in many physiological scenarios[23]. Eq. 1 simplifies to a system of linear first order differential equations[14]:

(2) $$\begin{array}{l} \frac{dx_{1}}{dt}=k_{1}^{+}f\left(t\right)-k_{1}^{-}x_{1}\\ \frac{dx_{i}}{dt}=k_{i}^{+}x_{i-1}-k_{i}^{-}x_{i} \end{array},$$

where the first species is activated at a rate $k_{1}^{+}$*f*(*t*); $k_{i}^{+}=\frac{x_{i}^{T}k_{cat}^{i}}{K_{M,i}}$ and $k_{i}^{-}=\frac{E_{pase}k_{cat,pase}^{i}}{K_{M,pase}^{i}}$. This scheme is depicted in Fig. 2a.

The weakly activated cascade model has the advantage that the linearity of the equations allows for analytical tractability. Eq. 2 can be conveniently analyzed by introducing Fourier transformed variables: *X*_*i*_(*ω*) = ∫*dte*^*iωt*^*x*_*i*_(*t*) and *F*(*ω*) = ∫*dte*^*iωt *^*f*(*t*). The number of activated species at stage *i *becomes $X_{i}\left(\omega \right)=\frac{k_{i}^{+}X_{i-1}\left(\omega \right)}{i\omega +k_{i}^{-}}$. The power spectrum *P*_*i*_(*ω*) ≡ |*X*_*i*_(*ω*)|^2 ^at the *i*^*th *^step can also be obtained: $P_{i}\left(\omega \right)=\frac{{\left(k_{i}^{+}\right)}^{2}}{\omega^{2}+{\left(k_{i}^{-}\right)}^{2}}P_{i-1}\left(\omega \right)$. After iterating at each successive stage of the *n *step cascade, an expression for *P*_*n*_(*ω*) as a function of the power spectrum of the input signal (*S*(*ω*) ≡ |*F*(*ω*)|^2^) is obtained:

(3) *P*_*n*_(*ω*) = *g*_*n*_(*ω*)*S*(*ω*),

in which a frequency dependent gain *g*_*n*_(*ω*) is defined as:

(4) $$g_{n}\left(\omega \right)=\prod_{i=1}^{n}{\left(\frac{k_{i}^{+}}{k_{i}^{-}}\right)}^{2}\frac{1}{1+{\left(\frac{\omega }{k_{i}^{-}}\right)}^{2}}.$$

*g*_*n*_(*ω*) is a transfer function that converts the input *S*(*ω*) into a response and provides a measure of the signal processing capabilities of the network. The change in the amplitude of the signal output is determined by the $\prod_{i=1}^{n}{\left(\frac{k_{i}^{+}}{k_{i}^{-}}\right)}^{2}$ term and the time dependence of the output is modulated by the ${\prod_{i=1}^{n}\left[1+{\left(\frac{\omega }{k_{i}^{-}}\right)}^{2}\right]}^{-1}$ term.

From the formula of *g*_*n*_(*ω*), one consequence of having multiple stages is readily apparent. In the high frequency regime $\left(\frac{\omega }{k_{i}^{-}}>>1\right)$, for each $k_{i}^{-}$, *g*_*n*_(*ω*) rapidly decays with increasing *n *(*g*_*n*_(*ω*) ~ *ω*^-2*n*^). Thus, longer cascades are more efficient at filtering the high frequency components of the signal from the output. This behavior is illustrated in Fig. 2b. Fig. 2b contains plots of *g*_*n*_(*ω*) for different values of *n*; cascades of lengths *n *= 1, 2, 3, 4 are shown.

In eq. 4, the relative values of $k_{i}^{-}$ along different stages of the cascade also affects the scaling behavior of *g*_*n*_(*ω*) as *ω *changes as well as the overall amplitude. The change in signal amplitude at the steady state (that leads to amplification or attenuation) at step *i *is given by the ratio of the effective rate constants for activation and deactivation $\frac{k_{i}^{+}}{k_{i}^{-}}$. $\frac{k_{i}^{+}}{k_{i}^{-}}>1$ results in signal amplification and $\frac{k_{i}^{+}}{k_{i}^{-}}<1$ leads to attenuation of the signal at step *i *[13,14]. Amplification or attenuation also leads to different time dependent behaviors of *g*_*n*_(*ω*). For example, consider an n staged cascade with rate constants $k_{i}^{-}$ such that $k_{n}^{-}>k_{n-1}^{-}>k_{n-2}^{-}...>k_{1}^{-}$, at frequencies *ω *> $k_{n}^{-}$, *g*_*n*_(*ω*) ~ *ω*^-2*n *^while at frequencies $k_{n}^{-}$ > *ω *> $k_{n-1}^{-}$, *g*_*n*_(*ω*) ~ *ω*^-2(*n*-1) ^and so forth. Thus, at intermediate frequencies, a time scale separation (as determined by different deactivation rates along the cascade), along with signal amplification and attenuation, also leads to different frequency dependent behaviors of *g*_*n*_(*ω*).

Also, from the plots in Fig. 2c (and inspection of eq. 4), it is observed that incorporation of faster steps along the cascade influences the frequency dependence of *g*_*n*_(*ω*) to a lesser extent than would be the case when the kinetics of activation are same for each successive step. Since signal propagation in these cases is limited by the slower stages of the cascade, the faster steps are effectively removed from *g*_*n*_(*ω*). This observation suggests a principle in the ability of a biochemical pathway to filter signals of short duration: when there is a positive gradient of deactivation rates (that also leads to amplification or attenuation of signal amplitude), the time dependence of signal integration for multi staged cascades more closely resembles that of a pathway involving a single step. This effect is a result of a single dominant time scale in the pathway and provides a mechanism for regulating the amplitude of the signal output while keeping the time dependence of the output the same as that of a single-staged pathway.

### Detection of short duration signals

While the sequential activation of multiple steps in a biochemical pathway allows for effective filtering of the high frequency, short duration components of a signal, often the desired signal output is regulated by feedback. We will now show with our analysis that feedback control in some instances also allows for the filtering of the low frequency, long duration components of a signal. Previous work has characterized this behavior with numerical simulations [17,18]. In these instances, signals that occur at short times can be integrated while signals with a longer duration are effectively filtered because at longer times, the negative feedback loop affects the signal output.

For instance, the signal output can be affected by a feedback loop that is initiated downstream of the output. This scenario would be the case when the signal output from a biochemical cascade activates its own positive or negative regulators. For instance, in mammalian cells, the activation of extracellular regulatory kinase (ERK) often leads to the upregulation or activation of its own phosphatases[24]. In this scenario, a signal output in the form of phosphorylated ERK (ERK is known to phosphorylate on the order of one hundred substrates) is deactivated as a result of the upregulation of phosphatases that dephosphosphorylate residues in the TxY motif whose phosphorylation is necessary for activation.

**Figure 3:**
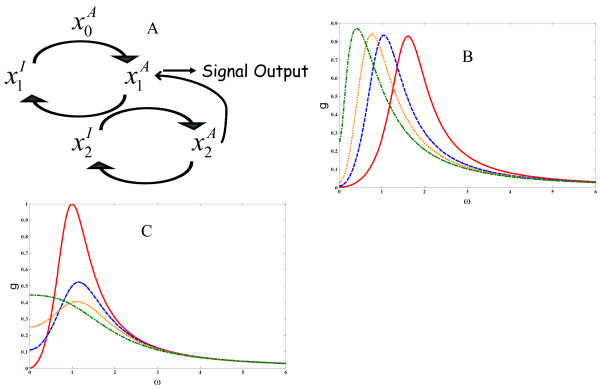
**Filtering of low frequency signals**. A three tiered biochemical cascade with competing processes occurring at different time scales that is sufficient to filter signals at long time scales (Low pass filtering). a.) An initial stimulus activates a downstream species that confers a signal output and activates a downstream species that, through feedback, interacts with the species that carries the signal output. Superscripts (I) and (A) denote inactive and active forms of each chemical species and are omitted from the equations in the text. b.) parameter values were taken to be: *υ *= -1, $k_{1}^{+}=k_{1}^{-}=k_{2}^{+}=1.0$; $k_{2}^{-}$ = 0.1. *k*_*f *_= 2.5 (solid lines), *k*_*f *_= 1.0 (dashed lines), *k*_*f *_= 0.5 (dotted lines), *k*_*f *_= 0.1 (dash-dotted lines). c.) parameter values were taken to be: *υ *= -1, $k_{1}^{+}=k_{1}^{-}=k_{2}^{+}=k_{f}=1.0$. $k_{2}^{-}$ = 0.0 (solid lines), $k_{2}^{-}$ = 0.5 (dashed lines), $k_{2}^{-}$ = 1.0 (dotted lines), $k_{2}^{-}$ = 2.0 (dash-dotted lines).

Signal output is the activity of the kinase at the *m*^*th *^step and feedback control to the signal output at step *m *is initiated at a later step (i.e. *n *> *m*) and the modified set of dynamical equations becomes:

(5) $$\begin{array}{l} \frac{dx_{1}}{dt}=k_{1}^{+}f\left(t\right)-k_{1}^{-}x_{1}\\ \frac{dx_{m}}{dt}=k_{m}^{+}x_{m-1}-k_{m}^{-}x_{m}+\upsilon k^{f}x_{n} \end{array}$$

where *k*^*f *^is the feedback strength and sets the time scale of the feedback and $\upsilon =\left\{\begin{array}{c} 1;positive\;feedback\\ -1;negative\;feedback \end{array}\right\}$. This scheme is depicted in Fig. 3a.

Another biologically important example involves a negative feedback loop that acts upon a downstream layer in the cascade. However, in the linear cascade approximation that is used in this paper (that allows for extensive mathematical analysis) such a feedback loop would simply act to effectively increase the rate of deactivation of the species involved in the layer of the cascade that is involved in the feedback interaction. A model with nonlinear negative feedback (and one that is not analytically tractable) would be required to show the effect mathematically.

After applying a Fourier transformation as before, an expression for $P_{m}^{f}(\omega )$ from eq. 5, albeit now more complicated, can be obtained as a function of *S*(*ω*) in closed-form:

(6) $$P_{m}^{f}\left(\omega \right)=g_{m}^{f}\left(\omega \right)S\left(\omega \right),$$

where,

(7) $$\begin{array}{c} g_{m}^{f}\left(\omega \right)=\prod_{i=1}^{m}\frac{{\left(k_{i}^{+}\right)}^{2}}{\left({\left(k_{i}^{-}\right)}^{2}+\omega^{2}\right)}\\ \begin{array}{l} +\frac{{\left(k^{f}\right)}^{2}\left[\prod_{i=1}^{n}{\left(k_{i}^{+}\right)}^{2}\right]}{\left({\left(k_{m}^{-}\right)}^{2}+\omega^{2}\right)\prod_{i=1}^{n}\left({\left(k_{i}^{-}\right)}^{2}+\omega^{2}\right)+{\left(k^{f}\right)}^{2}\prod_{i=m+1}^{n}{\left(k_{i}^{+}\right)}^{2}\prod_{i=1}^{m-1}\left({\left(k_{i}^{-}\right)}^{2}+\omega^{2}\right)+2\prod_{i=m}^{n}{\left(k_{i}^{+}\right)}^{2}\prod_{i=1}^{m-1}\left({\left(k_{i}^{-}\right)}^{2}+\omega^{2}\right)}\\ +\frac{\upsilon }{2}\frac{k^{f}\left[\prod_{i=1}^{n}\left(k_{i}^{+}\right)\right]\left[\prod_{i=1}^{m}\left(k_{i}^{+}\right)\right]}{\left[\prod_{i=m}^{n}\left(k_{i}^{-}\right)\right]\prod_{i=1}^{m}\left({\left(k_{i}^{-}\right)}^{2}+\omega^{2}\right)-\upsilon k^{f}\left({\left(k_{m}^{-}\right)}^{2}+\omega^{2}\right)\prod_{i=m+1}^{n}\left(k_{i}^{+}\right)\prod_{i=1}^{m-1}\left({\left(k_{i}^{-}\right)}^{2}+\omega^{2}\right)} \end{array} \end{array}$$

In the case of feedback regulation, there exists a competition between processes that are realized on multiple time scales: one for the signal to propagate along the cascade to the species involved in the signal output, and the others for the additional interactions derived from the feedback loops to propagate and interact with the species involved in the output. The competition between these effects in principle may lead to a frequency dependent optimal value of $g_{m}^{f}(\omega )$. At high frequencies as before, signal propagation is limited by the time it takes to move through the cascade and high frequency components of $g_{m}^{f}(\omega )$ are filtered. Also, at low frequencies, signals can potentially be attenuated when the response is dominated by the activity of the feedback loop. If the interaction from the feedback is sufficiently strong, then the low frequency components of the signal are also filtered by the cascade. In this scenario, the frequency dependent behavior of $g_{m}^{f}(\omega )$ would be non monotonic.

We illustrate these ideas through consideration of a three tiered cascade that consists of a chemical species carrying the input signal, a species conferring the signal output, and a species activated downstream to the output that provides a feedback interaction to the species conferring the signal output. In this scheme, *m *= 1 and *n *= 2, and eq. 7 is simplified and $g_{1}^{f}(\omega )$ becomes:

(8) $$g_{1}^{f}\left(\omega \right)=\frac{{\left(k_{1}^{+}\right)}^{2}\left[{\left(k_{2}^{-}\right)}^{2}+\omega^{2}\right]}{{\left(k_{1}^{-}k_{2}^{-}-\upsilon k_{f}k_{2}^{+}\right)}^{2}+\omega^{2}\left({\left(k_{1}^{-}\right)}^{2}+{\left(k_{2}^{-}\right)}^{2}+2\upsilon k_{f}k_{2}^{+}+\omega^{2}\right)}.$$

The optimal frequency *ω*_*opt *_is obtained by differentiating $g_{1}^{f}(\omega )$,

(9) $$\omega_{opt}={\left[{\left\{k_{f}k_{2}^{+}\left(k_{f}k_{2}^{+}-2\upsilon k_{2}^{-}\left(k_{1}^{-}+k_{2}^{-}\right)\right)\right\}}^{1/2}-{\left(k_{2}^{-}\right)}^{2}\right]}^{1/2}.$$

*ω*_*opt *_increases monotonically for decreasing values of $k_{2}^{-}$ and increasing values of *k*_*f*_. For negative feedback *υ *= -1, *ω*_*opt *_exists (Im *ω*_*opt *_= 0) when ${\left[k_{f}k_{2}^{+}\left\{k_{f}k_{2}^{+}+2k_{2}^{-}\left(k_{1}^{-}+k_{2}^{-}\right)\right\}\right]}^{1/4}>k_{2}^{-}$. Positive feedback *υ *= +1 requires that two conditions are satisfied for *ω*_*opt *_to be real, ${\left[k_{f}k_{2}^{+}\left(k_{f}k_{2}^{+}-2k_{1}^{-}k_{2}^{-}\right)\right]}^{1/4}>k_{2}^{-}$ and $k_{f}k_{2}^{+}>2k_{2}^{-}\left(k_{1}^{-}+k_{2}^{-}\right)$. The height at the optimal frequency $g_{1}^{f}(\omega_{opt})$ is:

(10) $$g_{1}^{f}\left(\omega_{opt}\right)=\frac{{\left(k_{1}^{+}\right)}^{2}}{{\left(k_{1}^{-}\right)}^{2}-{\left(k_{1}^{-}\right)}^{2}+2\left\{k_{f}k_{2}^{+}+\sqrt{k_{f}k_{2}^{+}\left[k_{f}k_{2}^{+}-2k_{2}^{-}\left(k_{1}^{-}+k_{2}^{-}\right)\right]}\right\}}.$$

We can also compute the width *ω*_1/2 _of $g_{1}^{f}(\omega )$ at half maximum $g_{1}^{f}(\omega_{opt})/2$. *ω*_1/2 _has the form:

(11) $$\omega_{1/2}=\frac{1}{\sqrt{2}}\left[\sqrt{4\gamma_{1}+\gamma_{2}+\sqrt{8\gamma_{1}\left(2\gamma_{1}+2\gamma_{3}\right)+\gamma_{4}}}-\sqrt{4\gamma_{1}+\gamma_{2}-\sqrt{8\gamma_{1}\left(2\gamma_{1}+2\gamma_{3}\right)+\gamma_{4}}}\right],$$

Where $\gamma_{1}=\sqrt{k_{f}k_{2}^{+}\left[k_{f}k_{2}^{+}-2k_{2}^{-}\left(k_{1}^{-}+k_{2}^{-}\right)\right]}$, $\gamma_{2}={\left(k_{1}^{-}\right)}^{2}-3{\left(k_{2}^{-}\right)}^{2}+2k_{f}k_{2}^{+}$, $\gamma_{3}=\gamma_{2}+2{\left(k_{2}^{-}\right)}^{2}$, and $\gamma_{4}={\left(k_{1}^{-}+k_{2}^{-}\right)}^{2}{\left(k_{1}^{-}-k_{2}^{-}\right)}^{2}+4k_{f}k_{2}^{+}$. Fig. 3b considers plots of $g_{1}^{f}(\omega )$ for different feedback strengths *k*_*f*_. The curves in Fig. 3b illustrate changes in $g_{1}^{f}(\omega )$, $g_{1}^{f}(\omega_{opt})$, and *ω*_1/2_. Fig. 3c illustrates how $g_{1}^{f}(\omega )$, $g_{1}^{f}(\omega_{opt})$, and *ω*_1/2 _change for different values of $k_{2}^{-}$.

### Differential detection of long and short signals by two interacting species

In the previous sections, we illustrated how long and short duration signals can be differentially detected with different network structures. Alternative schemes for detecting long and short duration signals are also possible. Consider a mechanism that illustrates the time dependence of signal transduction involving the competition of two interacting products. One product is produced in greater amounts at one time scale and the other is produced in great amounts at a different time scale. Similar schemes have been investigated in the context of MAPK signaling and have shown to have many effects on integrated signal output[17,18,24,25]. For example, in Yeast MAPK pathways, the output of one pathway (e.g. the stress-induced MAPK HOG1 pathway), can inhibit the activity of the (mating response-induced MAPK FUS3 pathway) as has been previously shown [25].

**Figure 4:**
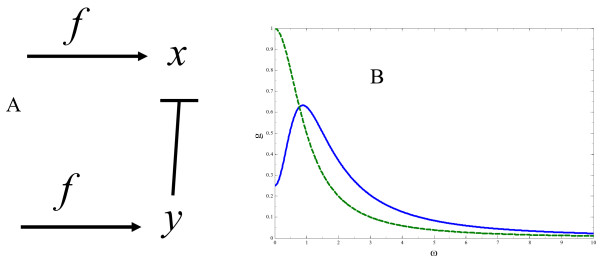
**A model of two interacting species produced by the same signal**. Differential time dependent signal detection by two competing products. a.) two species, denoted by X and Y in are produced by the same signal *f*(*t*). Species Y negatively interacts with species X. Species X can positively interact with itself. The activity of species f is transient with associated signal duration. b.) frequency dependent gain of two interacting products. Plots of *g*_*X*_(*ω*) (solid line) and *g*_*Y*_(*ω*) (dashed line) are shown. All parameters are taken to be 1.0 (in appropriate units) with the exception of *α*_1_; *α*_2 _= 1.5.

Here, we focus on the dynamics and time scale dependence of these mechanisms. Consider the following scheme depicted in Fig. 4a. In this scenario, two interacting products X and Y are produced by the same signal such as is the case of two parallel, interacting MAPK pathways. A set of two kinetic equations for species X and Y (denoted by *x *and *y *respectively) can be written as follows:

(12) $$\begin{array}{l} \frac{dx}{dt}=\alpha_{1}f\left(t\right)-\beta_{1}x-\gamma y\\ \frac{dy}{dt}=\alpha_{2}f\left(t\right)-\beta_{2}y \end{array}$$

*γ *is the strength of the negative interaction from species Y to species X. The other parameters that are introduced in this model are *α*_1 _and *α*_2_, that set the strength of interaction between the signal stimulus *f*(*t*) and X and Y respectively. The constitutive degradation rate constants of X and Y are *β*_1 _and *β*_2_.

We can solve eq. 12 using Fourier transformation as before. We define the following as before: *P*_*X*_(*ω*) ≡ |*X*(*ω*)|^2 ^and *P*_*Y*_(*ω*) ≡ |*Y*(*ω*)|^2^; where, $X\left(\omega \right)=\int_{-\infty }^{\infty }e^{i\omega t}x\left(t\right)$ and $Y\left(\omega \right)=\int_{-\infty }^{\infty }e^{i\omega t}y\left(t\right)$, and *S*(*ω*) = |*F*(*ω*)|^2 ^and $F\left(\omega \right)=\int_{-\infty }^{\infty }e^{i\omega t}f\left(t\right)$:

(13) *P*_*X*_(*ω*) = *g*_*X*_(*ω*)*S*(*ω*)

and

*P*_*Y*_(*ω*) = *g*_*Y*_(*ω*)*S*(*ω*).

The frequency dependent gain for species X and Y are obtained,

(14) $$g_{X}\left(\omega \right)=\frac{{\left(\alpha_{1}\right)}^{2}\left[{\left(\beta_{2}\right)}^{2}+\omega^{2}\right]+\gamma \alpha_{2}\left(\gamma \alpha_{2}-2\alpha_{1}\beta_{2}\right)}{\omega^{4}+\omega^{2}\left[{\left(\beta_{1}\right)}^{2}+{\left(\beta_{2}\right)}^{2}\right]+{\left(\beta_{1}\beta_{2}\right)}^{2}}$$

and

$$g_{Y}\left(\omega \right)=\frac{{\left(\alpha_{2}\right)}^{2}}{\omega^{2}+{\left(\beta_{2}\right)}^{2}}.$$

Plots of *g*_*X*_(*ω*) (solid) and *g*_*Y*_(*ω*) (dashed) are considered in Fig. 4b. From Fig. 4b, the following behavior is apparent. At very high frequencies, signals from *f*(*t*) are filtered by both products X and Y. At intermediate frequencies (*ω *~ 1.0 to *ω *~ 10.0) signals deriving from *f*(*t*) are integrated more efficiently by species X. Therefore, short duration signals are integrated more efficiently by species X. At low frequencies, *ω *< 1.0, signals originating from *f*(*t*) are integrated more efficiently by species Y. As a result, long duration signals are integrated more efficiently by species Y since at long times, X is affected by the negative interaction from Y.

This simple two species model illustrates how signal specificity can be achieved from two competing products by introducing changes in signal duration of the upstream signal. Short duration signals are more effectively integrated by one species and long duration signals are more effectively detected by the other.

### Integration of signals of differing duration

In the previous sections, we considered the frequency dependent gain of different network structures. In this section, we consider an incoming signal of differing duration and observe how it is differentially processed by networks that filter signals at different time scales. First, we considered the case in which the network filters short duration (high frequency) signals (Fig. 2). Next, we considered the case (Fig. 3) in which the network filters signals of long duration (low frequency).

**Figure 5:**
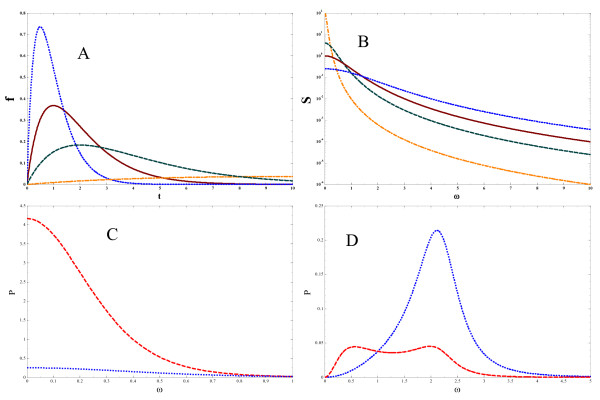
**Integration of differences in signal duration**. Differences in signal duration parameterized by *τ*_deg _a.) time domain. b.) frequency domain; $\tau_{\deg}^{-1}$ = 0.1 (dash-dotted), 0.5 (dashed), 1.0 (solid), 2.0 (dotted) lines. Plots of *S*(*ω*) ≡ |*F*(*ω*)|^2 ^are shown. Corresponding plots of *f*(*t*) are shown in the inset. Short $\tau_{\deg}^{-1}$ = 2.0 and long $\tau_{\deg}^{-1}$ = 0.5 duration signals are filtered through b.) *g*_3_(*ω*) and c.) $g_{1}^{f}(\omega )$ resulting in b.) *P*_3_(*ω*) and c.) $P_{1}^{f}(\omega )$ for $\tau_{\deg}^{-1}$ = 0.5 (dashed lines) and $\tau_{\deg}^{-1}$ = 2.0 (dotted lines). Parameters taken to be: b.) c) $k_{1}^{+}$ = 2.0, $k_{2}^{+}$ = 1.0, $k_{1}^{-}$ = 1.0, $k_{2}^{-}$ = 0.01, *k*^*f *^= -5.0.

A convenient way to parameterize signals of differing duration, while keeping the total amount of signal ∫ *f*(*t*)*dt *= *α *fixed, is to consider the function,

(15) $$f\left(t\right)=\alpha \frac{te^{-t/\tau_{d}}\Theta \left(t\right)}{\int te^{-t/\tau_{d}}\Theta \left(t\right)dt},$$

where Θ(*t*) is a Heaviside step function, *τ*_d _sets the signal duration, and *α *is taken to be 1 (*α *= 1) in the appropriate units. This form of *f*(*t*) models the behavior of a typical experimental signaling time course [4]. Plots of *f*(*t*) are shown in Fig. 5a. For this choice of signal, *S*(*ω*) is easily computed;

(16) $$S\left(\omega \right)=\frac{\alpha^{2}}{{\left(\tau_{d}^{-2}+\omega^{2}\right)}^{2}}.$$

*S*(*ω*) is plotted in Fig. 5b for different values of *τ*_d _ranging from $\tau_{\text{d}}^{-1}$ = 2.0 (short duration) to $\tau_{\text{d}}^{-1}$ = 0.1 (long duration).

Figs. 5c and 5d illustrate how signals *S*(*ω*) of large ($\tau_{\text{d}}^{-1}$ = 0.5 dotted lines) and small ($\tau_{\text{d}}^{-1}$ = 2.0 dashed lines) duration are integrated by the internal gains *g*_3_(*ω*) (Fig. 5c, from eq. 4) and $g_{1}^{f}(\omega )$ (Fig. 5d, from eq. 8) of these multistage cascades of differing network topologies. In Fig. 5c, the signal output *P*_3_(*ω*) (from eq. 3), upon integration by a three-tiered kinase cascade is shown. Taking $k_{i}^{+}=k_{i}^{-}=1.0$ for *i *∈ 1, 2, 3, *g*_3_(*ω*) effectively filters the short ($\tau_{\text{d}}^{-1}$ = 2.0) duration signal and results in an output *P*_3_(*ω*) of small magnitude at all time scales 2*πω*^-1 ^in the frequency spectrum. In contrast, for the signal characterized by $\tau_{\text{d}}^{-1}$ = 0.5, signal processing through *g*_3_(*ω*) results in a signal of larger amplitude. The ratio of amplitudes (with the superscript denoting the duration used) $\frac{P_{3}^{\tau_{d}=0.5}}{P_{3}^{\tau_{d}=2.0}}$ at the optimal frequency (*ω *= 0) for the two signals is $\frac{P_{3}^{\tau_{d}=0.5}}{P_{3}^{\tau_{d}=2.0}}$ ≈ 17.

In Fig. 5d, the signal output $P_{1}^{f}(\omega )$ (from eq. 6), obtained from a signal output that is also affected by a downstream negative (*υ *= -1) feedback loop, is shown. Parameters used are: $k_{1}^{+}$ = 2.0, $k_{2}^{+}$ = 1.0, $k_{1}^{-}$ = 1.0, $k_{2}^{-}$ = 0.01, *k*^*f *^= -5.0. For the signal of long duration $\tau_{\text{d}}^{-1}$ = 0.5, only the small frequency components of the signal are integrated. This behavior is in contrast with the signal output of a short duration signal $\tau_{\text{d}}^{-1}$ = 2.0. The amplitude difference in this case is $\frac{P_{1}^{f,\tau_{d}=0.5}}{P_{1}^{f,\tau_{d}=2.0}}$ ≈ 0.2.

## Discussion

Our models illustrate features of biochemical pathways that allow for the discrimination of signals that only differ in their duration. It is important to note that many important, nonlinear effects, at the expense of analytical tractability, were excised in making the linear, weakly activated cascade approximation. For example, nonlinear positive feedback is known to give rise to bistability. Also nonlinear negative feedback can lead to oscillatory behavior. These effects, however, correspond to long time, steady state behavior and the present analysis focused on transient signals of different duration and it is therefore expected that such nonlinear effects are not expected to influence the qualitative behavior of the results in this study.

In summary, we computed the frequency dependent internal gain for two classes of biochemical pathways involving multiple stages of regulation. The first model consisted of a cascade of steps and showed how changes in the number of steps as well as the amplification/attention of the signal changed the networks' ability to filter high frequency (short duration) components of a signal. Another network consisted of a sequence of steps in the form of biochemical intermediates in which the output is connected to a downstream feedback loop or an interacting product. The gain in this network can have non monotonic behavior in which the low frequency components of the signal are also filtered at time scales commensurate with the induction of the regulatory loop. This behavior enables the network to filter out signals of long duration. The minimal topological features of these biochemical networks provide distinct and robust mechanisms for integrating signals that persist with different characteristic time scales. As different temporally regulated signals often lead to different transcriptional programs such as in NF-κB signaling [26,27], it is tempting to speculate on the role that such filtering mechanisms may have in regulating gene expression.
